# Ascending Vagal Sensory and Central Noradrenergic Pathways Modulate Retrieval of Passive Avoidance Memory in Male Rats

**DOI:** 10.1002/jnr.25390

**Published:** 2024-10

**Authors:** Caitlyn M. Edwards, Inge Estefania Guerrero, Danielle Thompson, Tyla Dolezel, Linda Rinaman

**Affiliations:** Department of Psychology, Program in Neuroscience, Florida State University, Tallahassee, Florida, USA

**Keywords:** bed nucleus of the stria terminalis, conditioning, inhibitory avoidance, nucleus of the solitary tract, RRID:AB_10983245, RRID:AB_221448, RRID:AB_2245740, RRID:AB_2340813, vagus nerve, visceral feedback

## Abstract

Visceral feedback from the body is often subconscious, but plays an important role in guiding motivated behaviors. Vagal sensory neurons relay “gut feelings” to noradrenergic (NA) neurons in the caudal nucleus of the solitary tract (cNTS), which in turn project to the anterior ventrolateral bed nucleus of the stria terminalis (vlBNST) and other hypothalamic-limbic forebrain regions. Prior work supports a role for these circuits in modulating memory consolidation and extinction, but a potential role in retrieval of conditioned avoidance remains untested. To examine this, adult male rats underwent passive avoidance conditioning. We then lesioned gut-sensing vagal afferents by injecting cholecystokinin-conjugated saporin toxin (CSAP) into the vagal nodose ganglia (Experiment 1), or lesioned NA inputs to the vlBNST by injecting saporin toxin conjugated to an antibody against dopamine-beta hydroxylase (DSAP) into the vlBNST (Experiment 2). When avoidance behavior was later assessed, rats with vagal CSAP lesions or NA DSAP lesions displayed significantly increased conditioned passive avoidance. These new findings support the view that gut vagal afferents and the cNTS^NA^-to-vlBNST circuit play a role in modulating the expression/retrieval of learned passive avoidance. Overall, our data suggest a dynamic modulatory role of vagal sensory feedback to the limbic forebrain in integrating interoceptive signals with contextual cues that elicit conditioned avoidance behavior.

## 1 ∣ Introduction

Interoceptive information about the physiological state of the body is communicated continuously to the brain to shape current and future motivated behaviors (Maniscalco and Rinaman 2018). For example, visceral sensory signals arising in the gut can influence innate avoidance of stimuli that are naturally aversive (i.e., not requiring prior experience) (Maniscalco et al. 2015). In addition, our laboratory recently reported that visceral sensory feedback can influence avoidance of conditioned stimuli that become aversive after being paired with an innately aversive experience (Edwards, Dolezel, and Rinaman 2021; Edwards et al. 2023). Sensory feedback about the body's internal state is conveyed to the brain through blood-borne and neural pathways, the latter of which include vagal sensory pathways (Mayer 2011; Shapiro and Miselis 1985; Chen et al. 2021; Berntson, Sarter, and Cacioppo 2003; Ran et al. 2022). In one study, rats with selective destruction of vagal sensory afferents displayed less avoidance of innately aversive stimuli, but displayed prolonged freezing responses to an auditory conditioned fear cue (Klarer et al. 2014), suggesting that vagal sensory signaling can increase innate avoidance responses while reducing or suppressing learned fear responses. This discrepancy provides an interesting distinction between neural circuits that control innate avoidance/anxiety-like behavior and circuits that use experience and memory to generate conditioned fear responses. However, since sensory vagotomies in that study were performed prior to fear conditioning, it is unclear what aspect of fear learning was affected (i.e., acquisition, consolidation, retrieval, and/or extinction). To our knowledge, the necessity of vagal afferents and downstream circuits in the expression/retrieval of conditioned avoidance behavior has not been explored. While some research has examined the influence of vagus nerve stimulation on memory consolidation (Clark et al. 1998, 1995; Ghacibeh et al. 2009) and extinction (Noble et al. 2017; Peña et al. 2014; Alvarez-Dieppa et al. 2016), the potential role of vagal afferent signaling in the retrieval of passive avoidance memory is untested.

The central axon terminals of vagal viscerosensory afferents innervate postsynaptic neurons in the caudal nucleus of the solitary tract (cNTS), including neurons comprising the A2 noradrenergic (NA) cell group (Appleyard et al. 2007; Chen et al. 2020). cNTS^A2^ neurons project to a number of hypothalamic and limbic forebrain regions in which NA signaling modulates arousal and stress responses (Rinaman 2011). For example, cNTS^A2^ neurons densely innervate the anterior ventrolateral bed nucleus of the stria terminalis (vlBNST) (Phelix, Liposits, and Paull 1992; Terenzi and Ingram 1995), which receives additional NA input from the caudal ventrolateral medulla (A1 cell group) (Riche, De Pommery, and Menetrey 1990; Forray and Gysling 2004). A2/A1 neurons that innervate the vlBNST are recruited by a variety of acute innate stressors (Maniscalco et al. 2015; Rinaman 2011; Aston-Jones et al. 1999; Myers and Rinaman 2002; Zhu and Onaka 2002; Bienkowski and Rinaman 2013; Delfs et al. 1998; Dayas et al. 2001), and lesioning A2/A1 inputs to the vlBNST attenuates both innate stress-induced avoidance (Zheng and Rinaman 2013) and hypophagia (Banihashemi and Rinaman 2006). The vlBNST itself appears necessary for the expression of conditioned freezing responses to contextual fear stimuli (Zimmerman and Maren 2011; Sullivan et al. 2004), and has been implicated in other forms of conditioned behavioral suppression (Resstel et al. 2008; Waddell, Morris, and Bouton 2006). We recently reported that cNTS^A2^ neurons and neurons within the NA terminal-rich region of the anterior vlBNST are activated to express the immediate-early gene product, cFos, in rats exposed to a conditioned context that previously was paired with an aversive footshock (Edwards, Dolezel, and Rinaman 2021). The recruitment of these cNTS^A2^ and vlBNST neurons by a learned stressor implicates this ascending circuit in modulating conditioned avoidance behavior.

The present study was designed to test the hypothesis that vagal sensory afferents and the cNTS^NA^-to-vlBNST circuit modulate the expression of learned passive avoidance behavior. In *Experiment 1*, we sought to selectively lesion gastrointestinal vagal afferents by injecting cholecystokinin (CCK)-conjugated saporin toxin (CSAP) bilaterally into the nodose ganglia (Diepenbroek et al. 2017; McDougle et al. 2021). In *Experiment 2*, we sought to selectively lesion NA inputs to the anterior vlBNST by injecting saporin toxin conjugated to an antibody against dopamine-beta hydroxylase (DbH) (DSAP) bilaterally into the vlBNST (Zheng and Rinaman 2013; Banihashemi and Rinaman 2006; Rinaman and Dzmura 2007). In order to isolate lesion effects on the retrieval/expression of passive avoidance memory without impacting memory acquisition or consolidation, CSAP or DSAP lesions were made in rats *after* passive avoidance training.

## 2 ∣ Methods

All experiments were conducted in accordance with the National Institutes of Health *Guide for the Care and Use of Laboratory Animals* and were reviewed and approved by the Florida State University Animal Care and Use Committee.

### 2.1 ∣ Passive Avoidance Training and Testing

**FIGURE 1 ∣:**
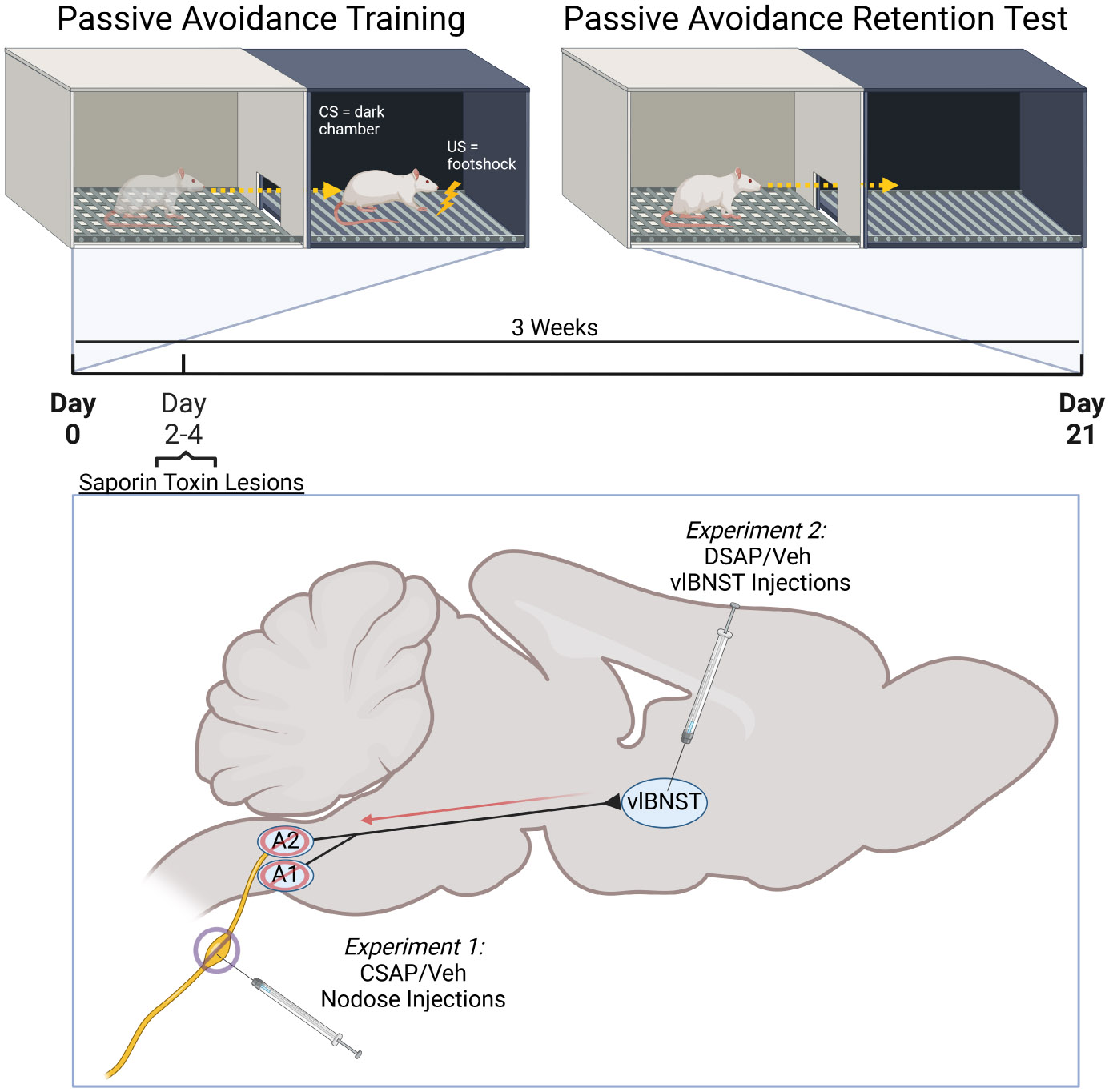
Experimental timeline depicting passive avoidance training, saporin toxin lesions, and passive avoidance retrieval testing. Rats trained in the passive avoidance paradigm learned to associate the dark chamber [conditioned stimulus (CS)] with a single mild electric footshock [unconditioned stimulus (US)]. Two to 4 days later, rats underwent a surgical procedure. In Experiment 1, CCK-conjugated saporin toxin (CSAP) or vehicle was injected into the nodose ganglia bilaterally to lesion gut-sensing vagal afferents. In Experiment 2, dopamine-beta hydroxylase (DbH) antibody-conjugated saporin toxin (DSAP) or vehicle was injected into the ventrolateral bed nucleus of the stria terminalis (vlBNST) bilaterally to lesion A2 and A1 noradrenergic (NA) neurons projecting to vlBNST. Three weeks later, passive avoidance of the US-paired dark chamber (i.e., latency to enter and time spent in the dark chamber) was quantified in each experiment.

Adult male Sprague Dawley rats (Envigo; 250–300 g body weight) were pair-housed in standard tub cages in a temperature- and light-controlled housing environment (lights on from 0400 to 1600 h). Rats were acclimated to handling for 3 days, with free access to water and rat chow (Purina 5001). Rats underwent passive avoidance training in a novel light/dark shuttle box (Colbourn Instruments, Allentown), with training performed 4–6 h after lights on (Figure 1). The light/dark box comprised a light (illuminated) chamber with clear plastic walls and a smooth white plastic floor, and a dark (nonilluminated) chamber with black plastic walls and a metal grid floor. Each chamber measured 25 × 25 cm, with 28 cm-high walls and a ceiling. The two chambers were separated by a metal divider wall with a guillotine door that could be opened and closed remotely. For passive avoidance training, rats were initially placed individually into the light chamber of the box and the dividing guillotine door was lifted immediately to allow access to the dark chamber. As expected, due to their innate aversion to the light and preference for the dark, rats very quickly entered the dark chamber (average latency = 19.47 s). Upon entry into the dark chamber, the guillotine door was closed. After a 5 s delay, rats received a single mild electric footshock (0.6 mA; 1 s). Rats remained in the enclosed dark chamber for 30 s following footshock, and then were returned to their homecage. Cagemates were similarly trained on the same day.

At least 2 days following training, rats underwent surgery to lesion gut-sensing vagal afferents (*Experiment 1*) or brainstem NA neurons that innervate the vlBNST, (*Experiment 2*) as described below (*CSAP/DSAP Injections*). Three weeks after training (at least 2 weeks after surgery), rats in both experiments were tested for passive avoidance retention. For this purpose, rats were placed individually into the light chamber of the light/dark box, the guillotine door was opened, and the latency of rats to fully enter the dark chamber was recorded, with a pre-set maximum latency of 900 s (15 min). During the retention test, the guillotine door remained open and no footshock was administered. Rats were allowed to freely explore both the light and dark chambers during the 900 s test, during which the number of entries and total time spent within each chamber was recorded. After testing was complete, rats were returned to their home cages. Four rats in the DSAP experiment (*n* = 2/group) were excluded from analysis due to the incorrect GraphicState protocol being used during testing. Passive avoidance behavior videos were coded by an experimenter blinded to treatment group.

### 2.2 ∣ Elevated Plus Maze

At least 2 weeks following completion of the passive avoidance task, rats in *Experiment 1* were placed in the center of an elevated plus maze (EPM) 4–6 h after lights on to assess their exploratory and anxiety-like behavior (Walf and Frye 2007), as in our prior work (Zheng and Rinaman 2013). The EPM was elevated 90 cm above the floor and comprised two open and two closed arms (each 45 × 10 cm) extending from a common central platform (10 × 10 cm). Rats were allowed to freely explore the maze for 5 min, while their behavior was recorded by a video camera mounted above the maze. After testing was complete, rats were returned to their home cages. EPM videos were coded by an experimenter blinded to treatment group. Quantified data included the number of arm entries and the total time spent by rats in the anxiogenic open arms of the maze during the 5 min test.

### 2.3 ∣ Open Field Test

At least 2 days following EPM testing, rats in *Experiment 1* were placed into the corner of a novel 95 × 95 cm open field (OF) with a novel ceramic object with rounded contours located in the center 4–6 h after lights on to assess their locomotion and exploratory behavior, including time spent in the “anxiogenic” center of the OF. Rats were allowed to freely explore for 10 min, while their behavior was recorded by a video camera mounted above the arena. Behavior in the OF was analyzed using automated behavior analysis software (ANY-maze, Stoelting Co.). Locomotion was defined as the total distance traveled during the test. Anxiety-like behavior was interpreted based on time spent in the center zone of the field (~30 × 30 cm).

### 2.4 ∣ Nodose CSAP Injections

For the CSAP experiment (*Experiment 1*), we used a highly specific vagal afferent lesioning agent to target gastrointestinal vagal afferents that express the gene encoding the CCK-A receptor (*Cckar*) (Diepenbroek et al. 2017; Patterson, Zheng, and Berthoud 2002). The lesioning agent (CSAP) is a conjugate of sulfonated CCK and the ribosome-inactivating protein, saporin [CCK-SAP; Advanced Targeting Systems, Inc.]. Rats were anesthetized with intraperitoneal injection of a cocktail containing ketamine (100 mg/kg) and xylazine (10 mg/kg) and placed into a supine position. The left and right nodose ganglia were identified and isolated as previously described (Diepenbroek et al. 2017). CSAP was diluted to 250 ng/μL in 0.15M NaCl vehicle containing 0.1% Fast Green. CSAP (*N* = 11 rats), or Fast Green vehicle alone (*N* = 5 rats) was delivered into each nodose ganglion by pressure injection (1.0 μL per ganglion) using a pulled glass capillary attached to a micromanipulator. Rats were allowed to recover from surgery and were tested for passive avoidance retention at least 2 weeks after surgery to allow time for effective lesioning (Diepenbroek et al. 2017).

### 2.5 ∣ BNST DSAP Injections

As in our previous studies (Zheng and Rinaman 2013; Banihashemi and Rinaman 2006), we used a highly specific approach to lesion NA neurons projecting to the anterior vlBNST (*Experiment 2*). The lesioning agent (DSAP) is a conjugate of a mouse monocloncal antibody to dopamine-beta hydroxylase (DbH) and the ribosome-inactivating protein, saporin [Anti-DbH-SAP; Advanced Targeting Systems, Inc.]. Rats (*N* = 25) were anesthetized by inhalation of isoflurane (1%–3% in oxygen) and placed into a stereotaxic apparatus. The anterior vlBNST (located 0.35 mm posterior, 2.3 mm lateral, and 7.4 mm ventral to bregma) was targeted using a 10° angle from vertical to avoid passing through the lateral ventricle and dorsal BNST. DSAP was diluted to 40 ng/100 nL in 0.15M NaCl vehicle containing 0.05% CTB, which does not interfere with DSAP lesion efficacy (Zheng and Rinaman 2013). DSAP (*N* = 11 rats) or CTB vehicle alone (*N* = 10 rats) was delivered (200 nL bilaterally) into the avlBNST by pressure injection (20 nL/min for 10 min) using a Hamilton syringe driven by a motorized injector (Stoelting Co.). The syringe was left in place for 10 min after each injection. Rats were allowed to recover from surgery and were tested for passive avoidance retention at least 2 weeks later, which is sufficient for effective lesioning (Zheng and Rinaman 2013; Banihashemi and Rinaman 2006; Rinaman 2003; Ippoliti et al. 1992; Wrenn et al. 1996).

### 2.6 ∣ Histology

Rats were deeply anesthetized with pentobarbital sodium (39 mg/mL i.p., Fatal Plus Solution; Butler Schein) and transcardially perfused with saline (100 mL) followed by 4% paraformaldehyde (500 mL). Fixed brains were removed from the skull, postfixed overnight at 4°C, then cryoprotected in 20% sucrose. Brains were blocked and sectioned coronally (35 μm) using a Leica freezing-stage sliding microtome. Tissue sections were collected in six serial sets and stored at −20°C in cryopreservant solution (Watson et al. 1986) until immunohistochemical processing. Primary and secondary antisera were diluted in 0.1M phosphate buffer containing 0.3% Triton X-100 and 1% normal donkey serum.

For rats used in the nodose CSAP lesion experiment (*Experiment 1*), heads were postfixed overnight at 4°C after brain removal. Fixed nodose ganglia were then dissected out, extracted and cryoprotected in 20% sucrose. Nodose ganglia were sectioned at 20 μm using a Leica cryostat and transferred directly to charged microscope slides for RNAscope-based analysis of lesion extent.

### 2.7 ∣ Verification and Characterization of Nodose CSAP Lesion

Quantification of left and right nodose ganglia lesion extent included analysis of 2–3 sections through each nodose ganglia from each rat in the CSAP experiment (*Experiment 1*). Sections were processed to detect surviving (i.e., nonlesioned) neurons expressing mRNA for the CCK-A receptor (*Cckar*). For this, the Rn-CCK-AR probe (412091, Accession No. NM_012688.3) and RNAscope Fluorescent Multiplex Assay (323100, ACDBio, San Franscico, CA) were used. Sections were incubated in channel 1-specific HRP (323104) and Cy5-TSAP (1:1000, tyramine signal amplification plus, NEL744E001KT; PerkinElmer, Waltham, MA, USA). After RNAscope labeling, slides were subsequently incubated in mouse monoclonal antiserum against the neuronal marker HuC/D (1:2500; ThermoFisher Scientific; A-21271; RRID:AB_221448) followed by Cy3-conjugated donkey anti-mouse IgG (1:500; Jackson ImmunoResearch; RRID:AB_10983245). Sections were imaged at 20× using a Keyence microscope (BZ-X710). The number of HuC/D-positive neurons present within each section and the number containing labeled CCK-AR transcripts were counted, with counts normalized to derive cell density within each analyzed area. Count densities within the left and right nodose ganglia were then averaged across 2–3 analyzed sections in each rat. Cell density counts from three nodose ganglia could not be quantified due to ineffective extraction (CSAP: *n* = 2; Veh: *n* = 1). For comparison, left and right nodose ganglia from two nonmanipulated control rats were also processed and analyzed to reveal potential physical damage arising from CSAP or Veh injections.

### 2.8 ∣ Verification and Characterization of DSAP Lesion

One set of tissue sections from each rat in the BNST DSAP experiment (*Experiment 2*; with each set containing a complete rostrocaudal series of sections spaced by 210 μm) was incubated in a mouse monoclonal antiserum against DbH (1:50,000; Millipore, MAB308; RRID:AB_2245740) followed by biotinylated donkey anti-mouse IgG (1:500; Jackson ImmunoResearch; RRID:AB_2340813). Sections were then treated with Elite Vectastain ABC reagents (Vector) and reacted with diaminobenzidine (DAB).

Sections through the cNTS containing the A2 cell group and VLM containing the A1 cell group (~15.2–14.15 mm caudal to bregma) were imaged at 20× using a Keyence microscope (BZ-X710). The total number of DbH+ neurons were counted bilaterally in each region and averaged per section. Sections through the pontine locus coeruleus (LC; ~10–10.2 mm caudal to bregma) were imaged at 10×. Due to extremely dense DbH labeling in the LC, it was not possible to visualize and accurately count individual DbH+ cells. Instead, a region of interest (ROI) encompassing the DbH labeling in LC was drawn using ImageJ bilaterally and the area of each ROI was measured and averaged across four LC ROIs (equivalent to bilateral LC in two tissue sections per rat).

### 2.9 ∣ Statistics

Sample sizes were determined based on previous reports (Edwards, Dolezel, and Rinaman 2021; Edwards et al. 2023). Data were analyzed using GraphPad Prism 10.3. Measures of passive avoidance behavior during the retention test (i.e., latency to enter, time in the dark chamber, and number of entries into the dark chamber) and exploratory/anxiety-like behavior in the elevated plus maze (i.e., time in the open arms and number of open arm entries) and the open field test (i.e., time in the center and distance traveled) were analyzed using unpaired Student's *t*-tests. Data quantifying *Cckar*-expressing nodose ganglia neurons for the CSAP experiment and DbH immunolabeling (cell counts in cNTS and VLM, area measurements in LC) for the DSAP experiment were analyzed using unpaired Student's *t*-test. In addition, anatomical data were correlated with passive avoidance behavior within-subjects using linear regression. An alpha level of 0.05 (*p* ≤ 0.05) was used as the criterion for considering group differences to be statistically significant. Estimation statistics were used to report effect sizes for both passive avoidance behavior and verification of CSAP and DSAP lesions (Calin-Jageman and Cumming 2019; Ho et al. 2019).

## 3 ∣ Results

### 3.1 ∣ Experiment 1: CSAP Lesions

#### 3.1.1 ∣ Passive Avoidance Task

**FIGURE 2 ∣:**
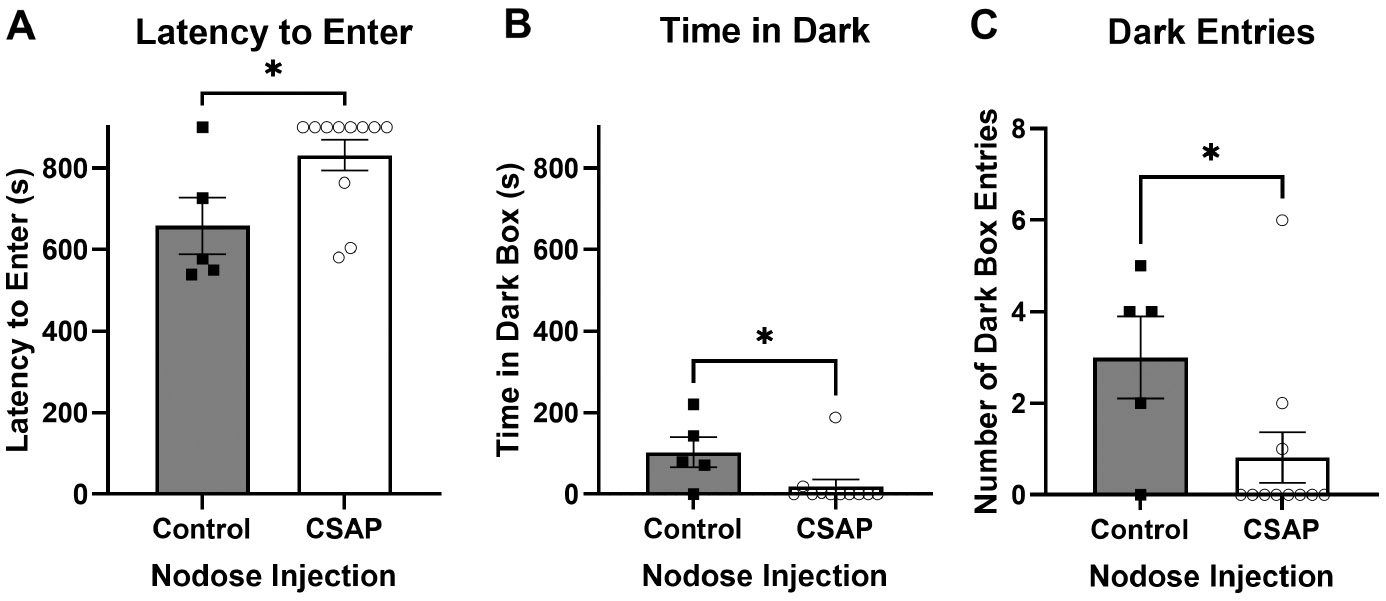
CSAP lesion increased passive avoidance behavior. (A) CSAP-lesioned rats took an average of 174 s longer to enter the shock-paired dark chamber compared with vehicle-injected controls [*t*(14) = 2.396; *p* = 0.0311]. (B) CSAP-lesioned rats spent an average of 84 s less in the shock-paired dark chamber compared with vehicle-injected controls [*t*(14) = 2.381; *p* = 0.0320]. (C) CSAP-lesioned rats entered the dark chamber an average of two times less compared with vehicle-injected controls [*t*(14) = 2.148; *p* = 0.0497]. *p < 0.05. Graphs display group mean ± standard error of the mean (SEM), with individual data points plotted.

Unpaired *t*-tests indicated that compared with vehicle-injected controls, CSAP-lesioned rats displayed more avoidance of the shock-paired dark chamber as measured by a longer latency to enter [*t*(19) = 2.396; *p* = 0.0311] (Figure 2A), less total time spent within the dark chamber [*t*(19) = 2.381; *p* = 0.0320] (Figure 2B), and fewer entries into the dark chamber [*t*(19) = 2.148; *p* = 0.0497] (Figure 2C). Passive avoidance behavior of one control rat that did not learn the task was excluded (latency to enter = 16 s). Examining the unpaired mean difference between CSAP- and vehicle-injected rats using estimation statistics indicated that CSAP rats took an average of 174 s (2.9 min) longer to enter the shock-paired dark chamber compared with vehicle controls [95% CI 5.64 s, 290 s] and spent an average of 83.5 s (1.4 min) less in the shock-paired dark chamber during the 900 s retention test [95% CI 19.2 s, 159 s].

#### 3.1.2 ∣ Elevated Plus Maze

**FIGURE 3 ∣:**
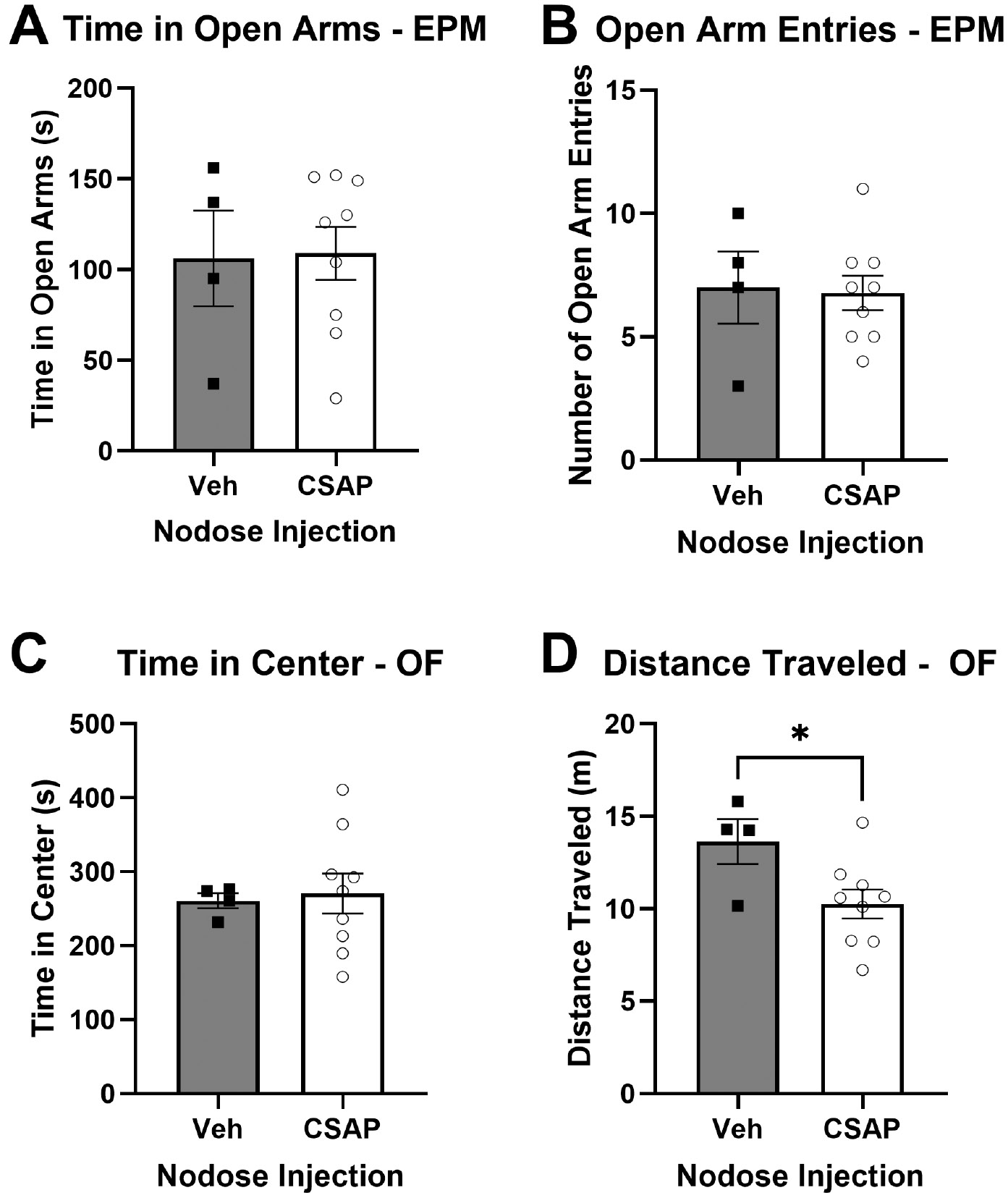
CSAP lesion does not affect innate anxiety-like behavior in the elevated plus maze (EPM) or the open field (OF), but reduces locomotion in the OF. (A) No differences were observed between groups in time spent in the open arms in the EPM [*t*(11) = 0.098; *p* = 0.9234]. (B) No differences were observed between groups in number of entries into the open arms in the EPM [*t*(11) = 0.1563; *p* = 0.8786]. (C) No differences were observed between groups in time in center of the OF [*t*(11) = 0.2346; *p* = 0.8189]. (D) CSAP-lesioned rats display less locomotion in the OF [*t*(11) = 2.366; *p* = 0.0374]. *p < 0.05. Graphs display group mean ± SEM, with individual data points plotted.

Unpaired *t*-tests indicated no significant differences between CSAP-lesioned and vehicle control rats in time spent in the open arms of the EPM [*t*(11) = 0.098; *p* = 0.9234] (Figure 3A) or in the number of open arm entries [*t*(11) = 0.1563; *p* = 0.8786] (Figure 3B).

#### 3.1.3 ∣ Open Field Test

Unpaired *t*-tests indicated no significant differences between CSAP-lesioned and vehicle control rats in time spent in the center of the OF [*t*(11) = 0.2346; *p* = 0.8189] (Figure 3C). Unpaired *t*-tests indicated a significant decrease in distance traveled in the OF for CSAP-lesioned rats compared to vehicle controls [*t*(11) = 2.366; *p* = 0.0374] (Figure 3D).

#### 3.1.4 ∣ Verification and Characterization of CSAP Lesion

One sample *t*-test indicated that the density of *Cckar*+ neurons in the left nodose of vehicle-injected and nonmanipulated rats did not significantly differ [*t*(4) = 1.215; *p* = 0.2912]. Conversely, the density of left nodose *Cckar*+ cells was significantly reduced compared with the nonmanipulated controls [*t*(8) = 14.63; *p* < 0.0001]. Similar results were seen in the right nodose ganglia, in which the density of *Cckar*+ neurons in vehicle-injected rats was similar to that in nonmanipulated rats [*t*(5) = 1.026; *p* = 0.3521], whereas the density of *Cckar*+ neurons in CSAP-injected rats was significantly lower than in nonmanipulated controls [*t*(8) = 5.894; *p* =0.0004].

**FIGURE 4 ∣:**
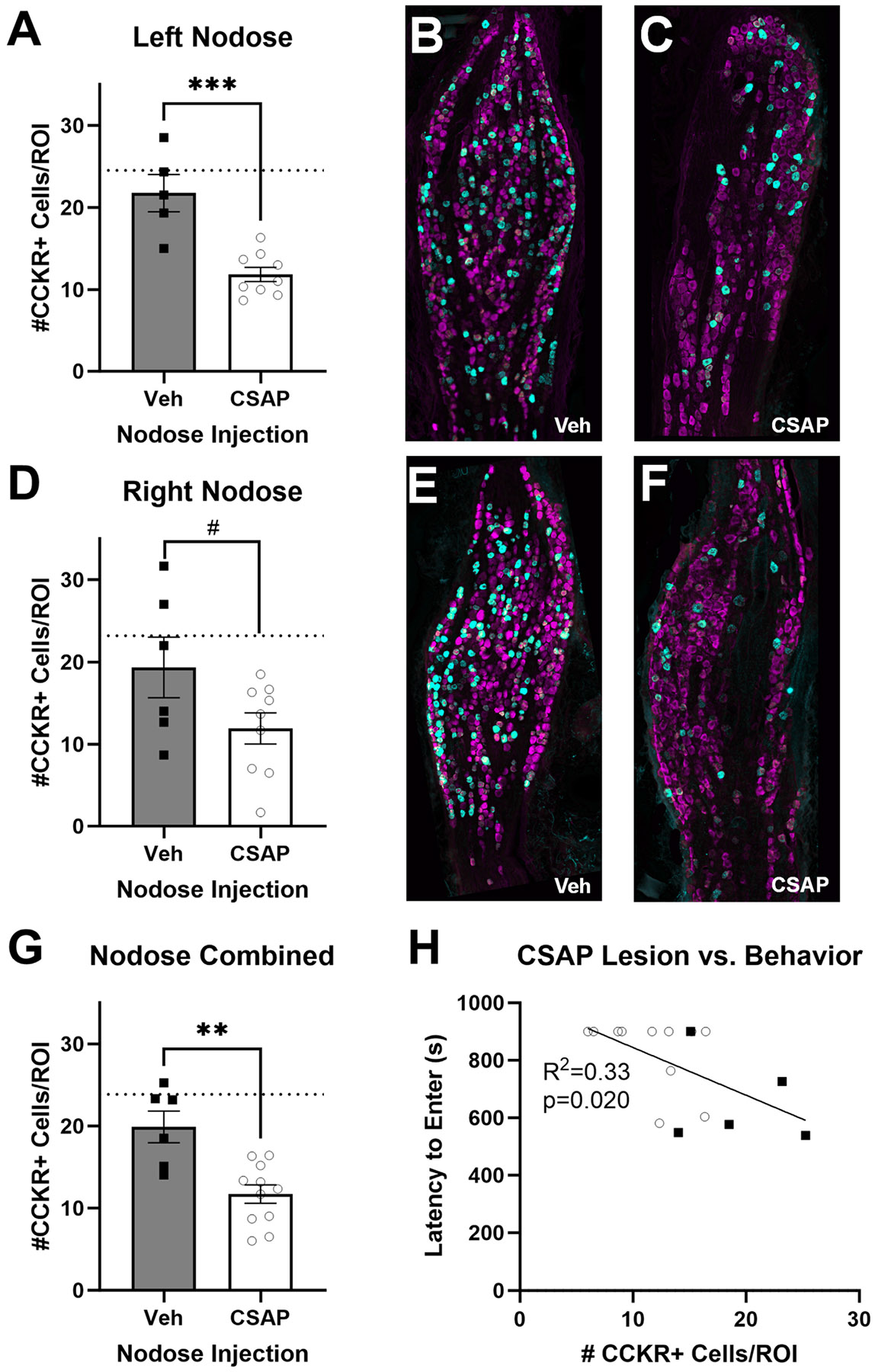
CSAP injection was effective at lesioning CCK-A receptor (*Cckar*)+ nodose ganglia neurons. (A) CSAP-lesioned rats displayed a 54.5% reduction in *Cckar*+ cells/region of interest (ROI) in the left nodose ganglia [*t*(12) = 4.890; *p* = 0.0004]. (B, C) Representative images of *Cckar*+ (blue) labeling and HuC/D (magenta) labeling in the left nodose ganglion of a vehicle control (B) and CSAP-lesioned rat (C). (D) Compared with vehicle-injected controls, CSAP-lesioned rats displayed 61.7% reduction in *Cckar*+ cells in the right nodose ganglia [*t*(13) = 1.967; *p* = 0.0709]. (E, F) Representative images of *Cckar*+ (blue) labeling and HuC/D (magenta) labeling in the right nodose ganglion of a vehicle control (E) and CSAP-lesioned rat (F). (G) Compared with vehicle-injected controls, CSAP-lesioned rats displayed 58.8% reduction in *Cckar*+ cells in the nodose ganglia combined [*t*(15) = 3.973; *p* = 0.0012]. (H) Degree of CSAP lesion is negatively related to the latency to enter the dark chamber [*t*(16) = −0.5760, *p* = 0.0195]. ^#^*p* < 0.10; **p < 0.01; ***p < 0.001. Graphs display group mean ± SEM, with individual data points plotted. Dotted lines on graphs represent the average of two noninjected nodose ganglia.

When data from rats with nodose vehicle injections were compared with data from CSAP-injected rats, unpaired *t*-test confirmed effective lesion of *Cckar*-expressing neurons in the nodose ganglia of CSAP-injected rats [*t*(15) = 3.973; *p* = 0.0012] (Figure 4G). Compared with vehicle-injected controls, the density of *Cckar*+ neurons was significantly reduced in the left nodose [*t*(12) = 4.890; *p* = 0.0004] (Figure 4A), with a trending reduction in the right nodose [*t*(13) = 1.967; *p* = 0.0709] (Figure 4D). Examining the unpaired mean difference between CSAP- and vehicle-injected rats using estimation statistics indicated that CSAP rats had an average of 40 fewer *Cckar*+ cells/mm^2^ in the left nodose ganglion [95% CI 22, 57] and 30 fewer *Cckar*+ cells/mm^2^ in the right nodose ganglion compared to vehicle-injected controls [95% CI −3, 62]. Unpaired *t*-test confirmed effective lesion when examining the number of *Cckar*+ neurons as a proportion to total number of HuC/D+ cells [*t*(15) = 3.246; *p* = 0.0054]. Compared with vehicle-injected controls, the proportion of *Cckar*+ HuC/D+ cells to the total number of HuC/D+ cells was significantly reduced in the left nodose [*t*(12) = 4.911; *p* = 0.0004], with a trending reduction in the right nodose [*t*(13) = 1.825; *p* = 0.0911].

We observed significant negative within-subjects relationships between the latency to enter the dark chamber and the extent of the CSAP lesion, including a negative correlation between the latency to enter and the number of *Cckar*+ cells in the combined left/right nodose ganglia [*r*(16) = −0.5760, *p* = 0.0195] (Figure 4H) and in the left nodose ganglion alone [*t*(13) = −0.5545, *p* = 0.0492] (Table 1). There was no significant correlation between latency to enter the dark chamber and the number of *Cckar*+ cells in the right nodose ganglion.

### 3.2 ∣ Experiment 2: DSAP Lesions

#### 3.2.1 ∣ Passive Avoidance Task

**FIGURE 5 ∣:**
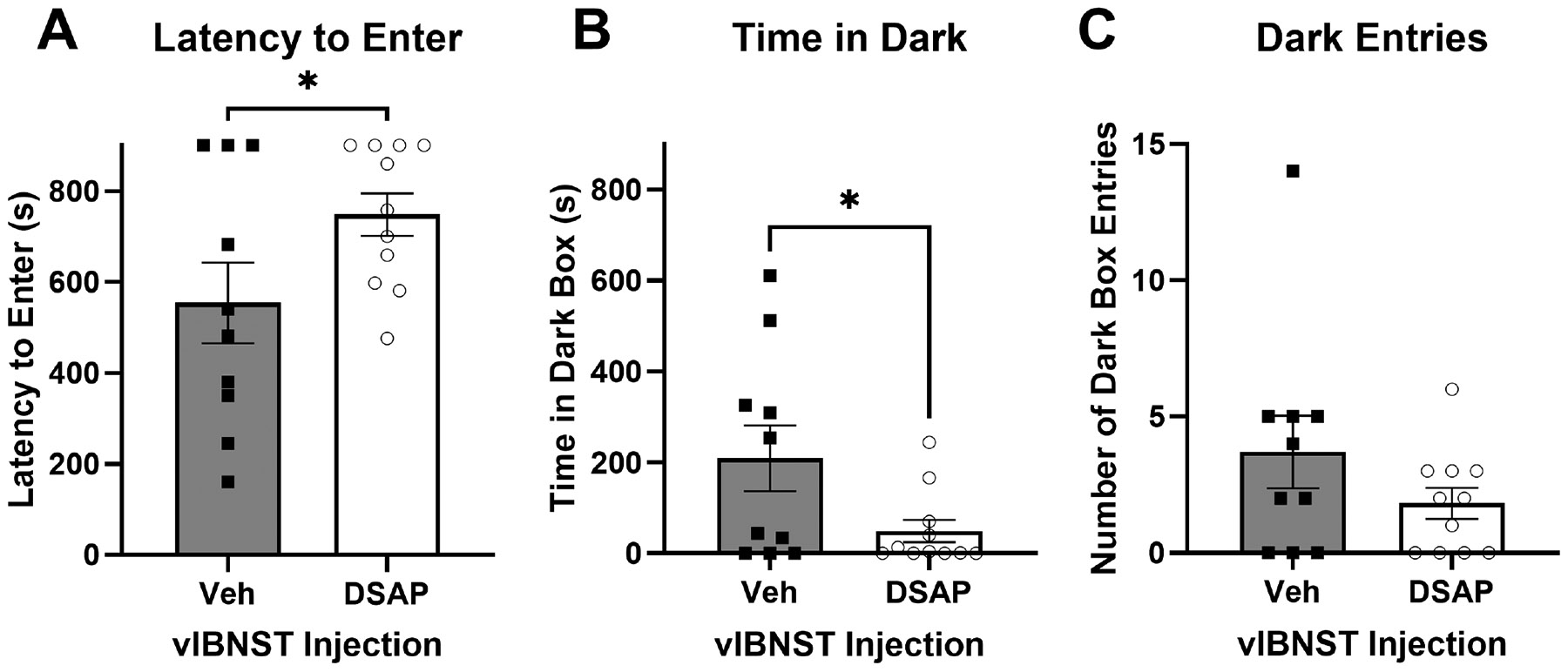
DSAP lesion increased passive avoidance behavior. (A) DSAP-lesioned rats took an average of 194 s longer to enter the shock-paired dark chamber than vehicle-injected controls [*t*(19) = 1.997; *p* = 0.0302]. (B) DSAP-lesioned rats spent an average of 160 s less in the shock-paired dark chamber than vehicle-injected controls [*t*(19) = 2.183; *p* = 0.0418]. (C) DSAP-lesioned rats do not display significant differences in number of entries into the dark chamber compared with vehicle-injected controls [*t*(19) = 1.349; *p* = 0.1933]. *p < 0.05. Graphs display group mean ± SEM, with individual data points plotted.

Unpaired *t*-test indicated that DSAP-lesioned rats displayed more avoidance of the shock-paired dark chamber as measured by a longer latency to enter [*t*(19) = 1.997; *p* = 0.0302] (Figure 5A) and less time spent in the dark chamber [*t*(19) = 2.183; *p* = 0.0418] (Figure 5B) compared to vehicle controls. There was no significant difference in the number of dark chamber entries between DSAP-lesioned rats and vehicle controls [*t*(19) = 1.349; *p* = 0.1933] (Figure 5C). Examining the unpaired mean difference between DSAP- and vehicle-injected rats using estimation statistics indicated DSAP rats took an average of 194 s (3.23 min) longer to enter the shock-paired dark chamber compared with vehicle controls [95% CI 4.08 s, 380 s] and spent an average of 160 s (2.67 min) less time within the shock-paired dark chamber during the 900 s retention test [95% CI 33.7 s, 322 s].

#### 3.2.2 ∣ Verification and Characterization of DSAP Lesion

**FIGURE 6 ∣:**
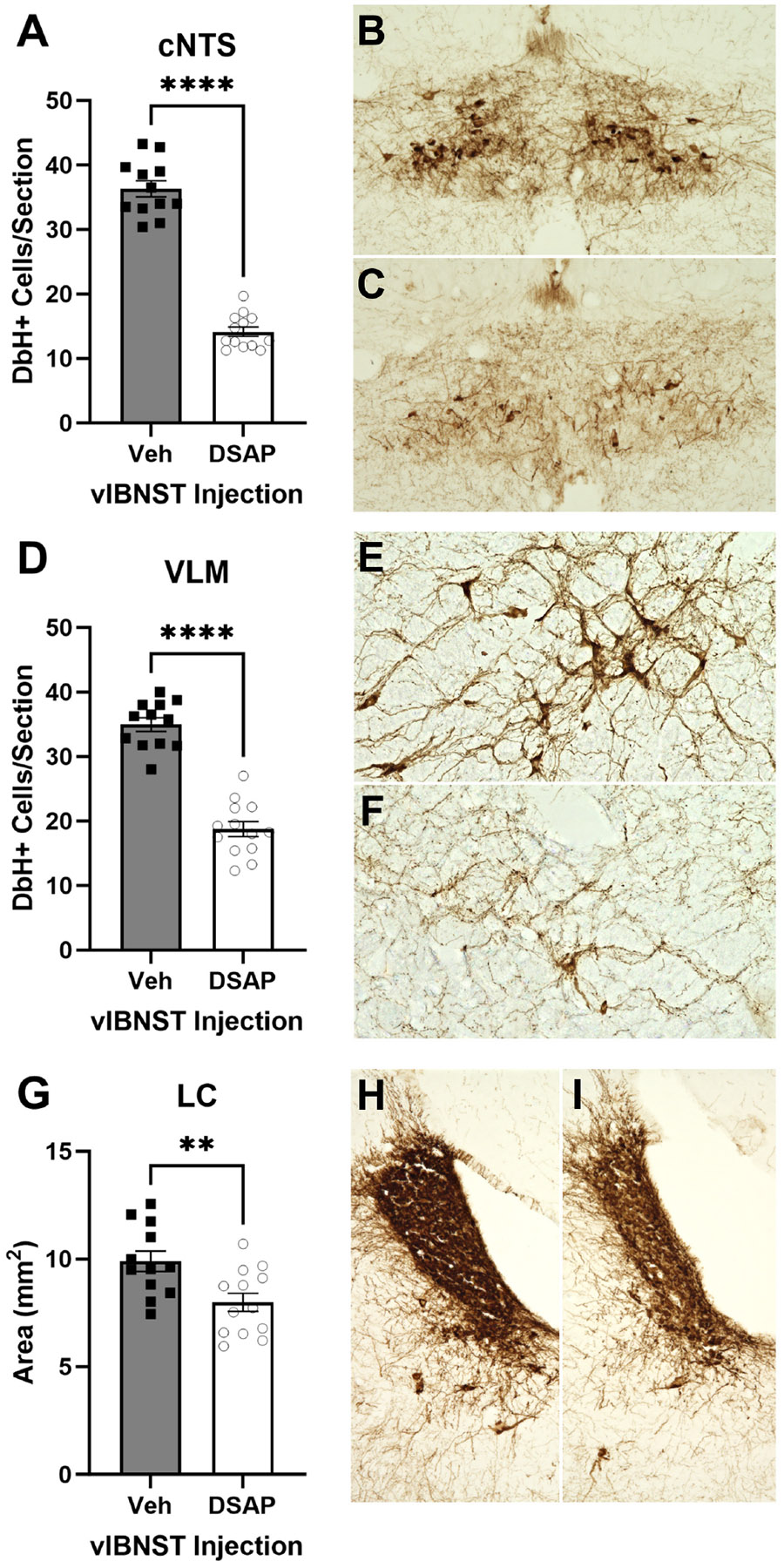
DSAP injection was effective at lesioning NA inputs. (A) Compared with vehicle-injected controls, DSAP-lesioned rats displayed a 60.1% reduction in DbH+ cells/section in the caudal nucleus of the solitary tract (cNTS) [*t*(23) = 15.63; *p* < 0.0001]. (B, C) Representative images of DbH+ labeling in the cNTS of a vehicle control (B) and DSAP-lesioned rat (C). (D) Compared with vehicle-injected controls, DSAP-lesioned rats displayed 47.1% reduction in DBH+ cells/section in the ventrolateral medulla (VLM) [*t*(23) = 10.30; *p* < 0.0001]. (E, F) Representative images of DbH+ labeling in the VLM of a vehicle control (E) and DSAP-lesioned rat (F). (G) Compared with vehicle-injected controls, DSAP-lesioned rats displayed 19.3% reduction in DBH+ staining area in the locus coeruleus (LC) [*t*(23) = 3.00; *p* = 0.0062]. (H, I) Representative images of DbH+ labeling in the LC of a vehicle control (H) and DSAP-lesioned rat (I). **p < 0.01; ****p < 0.0001. Graphs display group mean ± SEM, with individual data points plotted.

Unpaired *t*-test confirmed effective lesion of hindbrain DbH+ neurons. The number of DbH+ neurons per section was significantly reduced in both the cNTS [*t*(23) = 15.63; *p* < 0.0001] (Figure 6A) and the VLM [*t*(23) = 10.30; *p* < 0.0001] (Figure 6D). Examining the unpaired mean difference between DSAP- and vehicle-injected rats using estimation statistics indicated that DSAP rats had an average of 22.2 fewer DbH+ cNTS neurons per section [95% CI 19.5, 24.9] and 16.2 fewer DbH+ VLM neurons per section [95% CI 13, 18.9] compared to vehicle-injected controls, corresponding to an average loss of 60.1% and 47.1% of DbH+ neurons, respectively. Studen's *t*-test also revealed a moderate reduction in the area of DbH+ labeling within the LC in DSAP rats [*t*(23) = 3.00; *p* = 0.0062] (Figure 6G). Examining the unpaired mean difference between DSAP- and vehicle-injected rats using estimation statistics indicated that the average ROI area in DSAP rats (i.e., DbH+ LC area) was 1.91mm^2^ smaller than in vehicle-injected controls [95% CI 0.715, 3.09], a reduction in approximately 19.3%. We observed similar depletion of DbH+ labeling within the LC in previous studies in which DSAP was injected into the vlBNST (Zheng and Rinaman 2013), likely due to sparse axonal inputs from the LC to the vlBNST and closely adjacent areas.

We observed negative within-subjects relationships between the latency to enter the dark chamber and the extent of the DSAP lesion, including a trending negative correlation between the latency to enter and the number of DbH+ neurons/section in the cNTS [*r*(19) = −0.4201, *p* = 0.0580] (Table 2) and a significant negative correlation between the latency to enter and the number of DbH+ neurons/section in the VLM [*r*(19) = −0.4467, *p* = 0.0423] (Table 2). Conversely, there was no significant correlation between latency to enter the dark chamber and the area of DbH+ staining within the LC.

## 4 ∣ Discussion

Results from previous studies have implicated vagal afferent signaling and central noradrenergic signaling in avoidance behaviors in rodents (Klarer et al. 2014; Archer, Jonsson, and Ross 1985; Krypotos et al. 2015; Radwanska, Nikolaev, and Kaczmarek 2010). The present study is the first to demonstrate that expression of learned passive avoidance is modulated by vagal afferents and by noradrenergic neurons that provide axonal input to the vlBNST. Neurotoxin-induced loss of gut-sensing afferents in the vagus nerve (*Experiment 1*) or loss of noradrenergic inputs to the anterior vlBNST (*Experiment 2*) enhanced conditioned passive avoidance behavior in rats, as measured by their increased latency to enter and less time spent in the shock-paired dark chamber (Figures 2 and 5). The magnitude of these behavioral effects was closely related to the extent of loss of *Cckar*+ nodose neurons in CSAP-treated rats (Table 1) and of DbH+ neurons within the cNTS and VLM in DSAP-treated rats, but was not related to the more modest loss of DbH immunolabeling within the LC in DSAP-treated rats (Table 2).

It interesting to note distinctions between the effect of CSAP/DSAP lesions on innate and conditioned avoidance behaviors. We previously reported that similar DSAP-based lesioning of NA inputs to the vlBNST does not alter rats' innate avoidance of the open arms of the elevated plus maze (EPM) (Zheng and Rinaman 2013). Other laboratories report no effect of nodose ganglia CSAP lesion on innate avoidance of the open sections of the EPM or elevated zero maze (EZM) (Suarez et al. 2018; Krieger et al. 2022). In the current study, we replicated these findings by observing no effect of CSAP lesion on innate avoidance of the “anxiogenic” open arms of the EPM (Figure 3A,B) or the center of the open field (Figure 3C). Conversely, conditioned passive avoidance behavior was enhanced by CSAP lesions of vagal afferents in the nodose ganglia (Figure 2) or by DSAP lesions of NA neurons projecting to the vlBNST (Figure 5). These results support the view that vagal sensory and central NA circuits are differentially involved in innate anxiety-like behaviors vs. learned avoidance behaviors.

A handful of studies have lesioned gut-sensing vagal afferents to examine their role in memory. Our results appear to contrast with results from previous work in which vagal afferent lesions impaired memory in the hippocampal-dependent novel object-in-context test (Suarez et al. 2018), and also impaired flavor-nutrient learning (McDougle et al. 2024). It is possible that emotional valence associated with the memory task used here is a key factor. For example, vagal afferent signaling may suppress retrieval of negative emotional memories but may enhance retrieval of neutral or positive memories. Importantly, the prior studies cited above lesioned gut-sensing vagal afferents *prior to* training/learning, whereas CSAP lesions in our study were performed after learning had already taken place. Thus, the impact of nodose CSAP lesions on novel object (Suarez et al. 2018) and flavor memory (McDougle et al. 2024) may be to disrupt initial learning and/or memory consolidation.

Most published research investigating the influence of the vagus nerve on specific phases of memory has focused on manipulations during memory consolidation and extinction (Clark et al. 1998, 1995; Ghacibeh et al. 2009; Noble et al. 2017; Peña et al. 2014; Alvarez-Dieppa et al. 2016). Electrical and pharmacological stimulation of the vagus nerve posttraining (i.e., during memory consolidation) enhances passive avoidance memory in rodents (Clark et al. 1998, 1995). Interestingly, left cervical vagal nerve stimulation (VNS) in human patients during the consolidation phase immediately following learning enhances subsequent recognition memory (Clark et al. 1999). Consistent with our results indicating enhanced conditioned avoidance after vagal sensory lesions, VNS applied during retrieval *disrupts* memory performance in humans (Helmstaedter, Hoppe, and Elger 2001). Taken together, these data support the view that vagal afferent signaling plays a role in enhancing memory consolidation, while also contributing to disruption of memory retrieval. Much of the work examining the role of vagal afferent signaling in extinction has focused on learning related to posttraumatic stress disorder (PTSD) leveraging the idea of enhancing memory consolidation for extinction learning. Indeed, VNS delivered immediately following an extinction learning session enhances extinction of fear memory (Clark et al. 1998, 1995; Noble et al. 2017; Peña et al. 2014; Alvarez-Dieppa et al. 2016; Peña, Engineer, and McIntyre 2013; Szeska et al. 2020) and may be a promising treatment for PTSD (Szeska et al. 2020; George et al. 2008; Lamb et al. 2017; Wittbrodt et al. 2020), in which aberrant vagal signaling is considered to play a key role in symptomatology (Breit et al. 2018; McLaughlin, Alves, and Sheridan 2014). Given our current findings in which vagal afferents appear to play a role in suppressing fear memory during retrieval, it is possible that VNS may be an effective treatment for PTSD both through enhancing extinction and suppressing retrieval of traumatic memories.

The vagus nerve has been suggested to have indirect effects on memory by enhancing central noradrenergic signaling (Vonck et al. 2014). Previous studies have shown that brain-wide depletion of norepinephrine enhances spatial, contextual, and avoidance memory retrieval in rats (Kumar and Karanth 1994; Selden, Robbins, and Everitt 1990; Gheidi et al. 2023). Vagal afferents synapse directly onto A2 noradrenergic neurons in the cNTS (Appleyard et al. 2007; Chen et al. 2020), which provide the major source of noradrenergic input to the vlBNST (Aston-Jones et al. 1999; Fox et al. 2016). In *Experiment 2*, we demonstrate that lesioning noradrenergic inputs to the vlBNST enhances memory retrieval, providing circuit-specific insight regarding central sites at which norepinephrine depletion may influence memory retrieval. Thus, the vagal afferent and cNTS^NA^-to-vlBNST signaling pathways may serve to constrain or reduce fear memory retrieval, such that retrieval is enhanced when endogenous signaling through this pathway is interrupted by CSAP or DSAP lesions. It is important to note, however, that DSAP-induced destruction of NA neurons that innervate the vlBNST is accompanied by loss of other axon collaterals arising from the same NA neurons, which include axons in the hypothalamus (Bienkowski and Rinaman 2008). Loss of these additional NA pathways could contribute to the observed increase in conditioned passive avoidance behavior in DSAP rats.

An alternative interpretation of our experimental results is that the vagal afferent and cNTS^NA^-to-vlBNST pathways may be necessary to coordinate *active* (vs. passive) behavioral responses to threat, such that interruption of this circuit may bias the animal away from active and towards passive responses to threat. Consistent with this idea, while VNS reduces freezing behavior, a passive response to threat, VNS also has been shown to increase shuttling behavior in the active avoidance task (Slaughter and Hahn 1974). Furthermore, brain-wide norepinephrine depletion using DSP4 or global knockout of dopamine-beta hydroxylase (therefore reducing norepinephrine levels) have each been shown to reduce active avoidance learning (Archer, Jonsson, and Ross 1985; Krypotos et al. 2015; Radwanska, Nikolaev, and Kaczmarek 2010; Bennett et al. 1990; Thomas and Palmiter 1997). Additionally, the BNST specifically appears to play a similar role in biasing threat responses toward active coping behaviors, including active struggle bouts during restraint stress, which are associated with increased BNST neural activity (Luchsinger et al. 2021). Furthermore, chemogenetically inhibition of BNST neurons reduces active avoidance shuttling responses (Guerra et al. 2023). While our data are consistent with the hypothesis that the vagal afferent-to-cNTS^A2^-to-vlBNST circuit is necessary for active responses to threat, additional work will be needed to examine the role of gut-sensing vagal afferents and noradrenergic inputs to the vlBNST in active avoidance behavior.

The data presented here are the first to examine retrieval of conditioned passive avoidance behavior specifically in rats in which gut-sensing (*Cckar*-expressing) vagal sensory afferents or noradrenergic inputs to the vlBNST were lesioned after training had already occurred. The temporal and circuit specificity of these lesions allowed us to isolate our analysis on the role of these circuit components in passive avoidance memory retrieval, without interfering with earlier phases of learning and memory. It is important to note, however, that while these lesions were made after footshock training, they permanently destroyed the affected neuronal populations. Thus, compensatory neuroadaptations could have occurred during the 2-week postlesion period. In addition, the effects of partial chronic lesions such as those in the present study could differ from the effects of more transient or more complete interruption of vagal sensory and/or noradrenergic pathways. Future research should examine how more acute and reversible manipulations of neural activity in these ascending circuits influence passive avoidance retrieval.

In *Experiment 1, Cckar*-expressing neurons in the left nodose ganglia were more completely lesioned than *Cckar*+ cells in the right nodose ganglia. It is interesting to note that both preclinical and clinical studies as well as treatments utilizing VNS tend to administer stimulation unilaterally to the left vagus (Noller et al. 2019; Ogbonnaya and Kaliaperumal 2013). While no studies have examined the effect of VNS on passive avoidance retrieval, left VNS has been shown to reduce figural memory retrieval (Helmstaedter, Hoppe, and Elger 2001) and to reduce symptoms of anxiety in some human patients (George et al. 2008; Groves and Brown 2005). Since there is some indication of lateralized functions of the left versus right vagus nerve in mice and rats, particularly with the involvement of the right vagus nerve in reward (Han et al. 2018; Brougher et al. 2021), it would be interesting to examine whether laterality exists in vagal afferent pathways that modulate memory and avoidance.

Our data also indicate that CSAP lesions reduced locomotion in the open field test (Figure 3D). This result is important to note in the context of the passive avoidance behavior in which CSAP-lesioned rats took longer to enter the dark chamber than vehicle controls (Figure 2A). Thus, locomotion may have contributed to the increased amount of time it took for CSAP-lesioned rats to enter the dark chamber. However, these rats were not lethargic, as they traveled an average of 10.26 m during the 10-min open field test. Since the passive avoidance test took place in a small chamber over the course of 15 min, it is unlikely that locomotion deficits alone explain the increase in passive avoidance behavior after nodose CSAP lesions.

Female rats were not included in our current study based on our previous findings that passive avoidance behavior is displayed more strongly in male rats and that visceral feedback about metabolic state modulates passive avoidance in male but not female rats (Edwards, Dolezel, and Rinaman 2021). Previous work has demonstrated sex-specific effects of central norepinephrine depletion on spatial memory retrieval in the Barnes maze, such that norepinephrine depletion improves performance in males, but impairs performance in females (Gheidi et al. 2023). Therefore, it will be important in future work to examine the role of vlBNST-projecting noradrenergic inputs on passive avoidance memory retrieval in females.

Overall, results from the present study demonstrate that lesioning gut-sensing neurons in the nodose ganglia and noradrenergic neurons whose axons target the vlBNST enhances conditioned passive avoidance behavior in male rats. These new findings support the view that gut-sensing vagal afferents and hindbrain NA inputs to the vlBNST play a role in modulating the expression/retrieval of passive avoidance. Understanding how this circuitry contributes to conditioned avoidance may provide insight into effective treatment strategies for excessive avoidance behaviors in human anxiety and stress-related disorders.

## Supplementary Material

Transparent Science Questionnaire for Authors

Additional supporting information can be found online in the Supporting Information section.

## Figures and Tables

**TABLE 1 ∣:** Correlations between the CSAP lesion and behaviors in the passive avoidance task.

	Both nodose #*Cckar*+ cells/ROI	Left nodose #*Cckar*+ cells/ROI	Right nodose #*Cckar*+ cells/ROI
Latency to enter	*r* = −0.5760	*r* = −0.5545	*r* = −0.4259
	***p* = 0.0195** *	***p* = 0.0492** *	*p* = 0.1289
Time in dark	*r* = 0.3477	*r* = 0.4502	*r* = 0.2316
	*p* = 0.1869	*p* = 0.1227	*p* = 0.4063

*Note: r* = Pearson's correlation coefficient.

* Statistically significant or trending effects are highlighted in bold.

**TABLE 2 ∣:** Correlations between the DbH immunolabeling and behaviors in the passive avoidance task.

	DbH+ cells in cNTS	DbH+ cells in VLM	DbH+ area in LC
Latency to enter	*r* = −0.4201	*r* = −0.4467	*r* = −0.1143
	***p* = 0.0580** ^#^	*p* = 0.0423^*^	*p* = 0.6216
Time in dark	*r* = 0.4316	*r* = 0.4755	*r* = 0.1822
	***p* = 0.0508** ^#^	*p* = 0.0294^*^	*p* = 0.4292

*Note: r* = Pearson's correlation coefficient.

* Statistically significant or

# trending effects are highlighted in bold.

## Data Availability

All data generated or analyzed during this study are included in this published article. Further enquiries can be directed to the corresponding author.

## Summary

- Many anxiety and stress-related disorders are characterized by excessive learned avoidance.
- Our previous work demonstrates that feedback about internal state modulates retrieval of learned avoidance.
- We now extend those findings by demonstrating that gut-sensing vagal afferents and hindbrain noradrenergic inputs to the vlBNST contribute to avoidance memory retrieval and expression, such that damage to either circuit component enhances conditioned passive avoidance.
- Our results invite continued research focus on the structure and function of interoceptive signaling pathways and their modulatory role in shaping motivated behavior under normal and pathological conditions.

## Abbreviations:

CCK: cholecystokinin
Cckar: cholecystokinin A receptor
cNTS: caudal nucleus of the solitary tract
CSAP: cholecystokinin-conjugated saporin toxin
DbH: dopamine-beta hydroxylase
DSAP: dopamine-beta hydroxylase antibody-conjugated saporin toxin
EPM: elevated plus maze
LC: locus coeruleus
NA: noradrenergic
OF: open field test
ROI: region of interest
vlBNST: ventrolateral bed nucleus of the stria terminalis
VLM: ventrolateral medulla
VNS: vagal nerve stimulation
